# Social Determinants of Health and Quality of Life Among Korean Americans with Chronic Hepatitis B

**DOI:** 10.21203/rs.3.rs-8206683/v1

**Published:** 2025-12-26

**Authors:** Cindy Xin Fang, Yoonjin Nam, Ann C Klassen, Hee-Soon Juon

**Affiliations:** Thomas Jefferson University; Thomas Jefferson University; Drexel University; Thomas Jefferson University

## Abstract

**Background.:**

Hepatitis B virus (HBV) infection disproportionately affects Asian Americans, who often experience additional social and structural challenges that may influence quality of life. This study examined how social determinants of health (SDOH) are associated with physical and mental health related quality of life among Korean Americans living with chronic hepatitis B (CHB).

**Methods.:**

A total of 365 CHB patients completed the enrollment survey. SF-12 was used to measure quality of life, calculating physical and mental components summary scores (PCS-12 and MCS-12). SDOH were measured across five domains, including education access, economic stability, social/community context, neighborhood, and health care access. Descriptive statistics and multivariable linear regression were used.

**Results.:**

Participants had a mean age of 60.1 years, and 44% were female. In multiple linear regression analyses, employment and financial stability were significantly associated with higher PCS-12 and MCS-12 scores. Social support and perceived neighborhood social cohesion were associated with better mental health, while marital status was associated with better physical health. In addition, females had lower levels of physical health than males. Older adults reported worse physical but better mental health.

**Conclusions.:**

Economic stability, social support, and neighborhood cohesion are the key determinants of quality of life among Korean Americans with CHB. These findings emphasize the need for interventions that address both structural and psychosocial factors to improve quality of life in this underserved population. Further research is warranted to explore the nuanced dynamics of different SDOH domains interact over time and to identify intervention targets in this underserved population.

## INTRODUCTION

Viral hepatitis continues to be a global health threat and was the second leading cause of death among communicable diseases worldwide in 2022. Hepatitis B virus (HBV) infection accounted for an estimated 1.1 million deaths, representing 83% of all hepatitis deaths.[1] HBV is a hepatotropic DNA virus that can cause either acute or chronic infection. Chronic hepatitis B (CHB) can progressively lead to liver damage and eventually cirrhosis, which will substantially increase the risk of hepatocellular carcinoma (HCC), liver failure, and mortality [2]. According to the WHO global hepatitis report in 2024, approximately 254 million people are living with CHB, and CHB is responsible for more than 40% of cirrhosis and 60% of HCC cases worldwide [1].

In the United States, the burden of HBV and CHB disproportionately affects immigrant populations, particularly Asian Americans. A 2018 meta-analysis estimated that of the 1.89 million people living with CHB in the U.S., more than 75% (1.47 million) were born outside the country [3]. Among these, Asian Americans represent the highest-risk group, with a prevalence rate more than ten times that of non-Hispanic whites [4, 5]. Despite these alarming disparities, this population remains underrepresented in research and underserved in hepatitis B care.

In addition to these individual-level experiences, broader socioeconomic challenges, including financial instability, social marginalization, housing insecurity, and unemployment, may increase disease burden [6, 7]. These psychosocial burdens emphasize the need to monitor not only clinical outcomes but also health-related quality of life (HRQoL), a multidimensional construct encompassing physical, emotional, and social functioning, as a component of comprehensive CHB management.

Many evidence emphasizes the importance of social determinants of health (SDOH) as an important upstream driver of health disparities and quality of life. The U.S.’s Healthy People 2030 campaign has placed an “increased and overarching focus on social determinants of health,” which is crucial for the journey towards reducing health disparities and achieving health equity. SDOH is defined as “conditions in the environments where people are born, live, learn, work, play, worship, and age that affect a wide range of health, functioning, and quality-of-life outcomes and risks”. SDOH is categorized in five domains: 1) economic stability, 2) healthcare access and quality, 3) education access and quality, 4) social and community context, and 5) neighborhood and built environment [8].

SDOH factors have been shown to impact the development, progression, and management of chronic diseases, including chronic liver disease [9]. Existing literature suggests that financial strain has been associated with increased risk of liver disease progression to HCC and exacerbates the burden of long-term antiviral treatment in the CHB patient population [10–12]. Studies show that CHB patients with compensated liver disease incur nearly three times the healthcare costs of non-CHB patients, while those with more advanced disease face even higher all-cause annual healthcare costs across all insurance types. Notably, CHB patients with Medicare have significantly higher healthcare costs compared to those with commercial insurance ($69,107 vs. $18,618) [13]. These long-term treatment costs can contribute to economic hardship, especially for patients without sufficient insurance coverage or those living in low-resource communities.

Social support plays an important role in self-managing chronic diseases and promoting treatment adherence, including HIV, chronic kidney disease, chronic hepatitis, asthma, cardiovascular diseases, and diabetes [14–16]. In a study involving telephone interviews with CHB patients in North America, participants emphasized the importance of their support networks in reducing anxiety and alleviating mental distress by providing reassurance and a sense of being cared for. In contrast, limited social support has been linked with lower rates of CHB diagnosis and suboptimal patient care, particularly in the immigrant population [17]. The emotional and social burdens imposed by CHB may further hinder community and social integration, creating a cycle of disengagement from care and worsening their health outcomes. Marital status, a proxy for close interpersonal support, has been associated with better health outcomes and treatment adherence in chronic disease populations. Married individuals often report higher self-rated health and are more likely to engage in beneficial behaviors, such as improved diet and medication adherence [18–20]. However, the quality of the relationship also matters, as strained partnerships may diminish these potential benefits [21].

Educational attainment has also correlated with health behaviors and outcomes in chronic conditions, such as diabetes, hypertension, heart disease, chronic kidney disease, and COPD, but its relationship to CHB health outcomes remains underexplored [22–24]. Previous studies suggest that educational interventions have a beneficial impact on behavioral modifications of CHB patients, including HBV testing and treatment adherence [17, 25].

Neighborhood characteristics are a critical yet often understudied domain within the broader framework of SDOH, with increasing evidence linking them to chronic disease outcomes and overall well-being. Social Disorganization Theory suggests that deteriorating neighborhood physical and social environments, such as poor infrastructure, lack of safety, and weakened social cohesion, can contribute to social isolation, psychological distress, reduced healthcare access, and eventually adverse cognitive and physical health outcomes [26–29]. These effects are particularly prominent in vulnerable populations such as older adults and individuals with lower levels of educational attainment [26, 30, 31]. In the context of CHB, poor neighborhood environments may compromise CHB care engagement, treatment adherence, and mental health, which further exacerbate disparities in HRQoL.

Healthcare access and quality a particularly important SDOH domains for CHB and chronic liver disease patients. Appropriate screening, early diagnosis, and long-term antiviral therapy depend on continued access to care. Immigrant populations who carry a disproportionate burden of CHB particularly face significant barriers due to the complexity of the U.S. healthcare system, limited insurance coverage, and language barriers. Qualitative studies have shown that copayments exceeding patients’ financial capacity and changes to prescription plans can impede adherence to antiviral treatment and regular specialty care [32]. Additionally, misinformation about the need for long-term HBV care, low satisfaction with healthcare services, and hepatitis B-related stigma have been identified as additional barriers that discourage care-seeking behavior in Chinese American populations [33].

These broader SDOH factors are especially significant for CHB patients, many of whom are immigrants navigating multiple structural barriers. While prior studies have explored individual domains of SDOH, few have systematically examined how these factors influence both physical and mental dimensions of HRQoL in CHB populations. Even fewer studies have focused on high-burden and high-risk immigrant subgroups, such as Asian Americans and Korean Americans, who often face unique structural barriers to care.

To address this gap, this study aims to examine the relationships between five SDOH domains and both physical and mental components of HRQoL in Korean American adults with CHB. HRQoL in this study was measured using the 12-item Short Form Health Survey (SF-12), a widely validated instrument for evaluating physical and mental health functioning in the chronic disease population [34]. By exploring how specific SDOH domains are associated with patient-reported outcomes, this study aims to inform patient-centered, culturally sensitive strategies to improve quality of life among CHB patients. Moreover, many CHB patients require long-term antiviral therapy, so understanding how treatment intersects with social determinants of health and patient-reported outcomes can inform more equitable models of CHB care and guide the development of treatments that better reflect patients’ needs.

## METHODS

### Happy Life Healthy Liver (HL2) Cohort Study of chronic hepatitis B patients

The Healthy Liver Happy Life (HL2) cohort study of Korean American patients with CHB was established in two cities: Philadelphia, PA, and Los Angeles, CA. Full details on the study design and recruitment methods have been reported previously [35]. The study aimed to examine the bio-psychosocial models of liver disease progression in CHB patients receiving care from Korean American hepatologists. Recruitment activities targeted CHB patients who visited the clinics for regular follow-up visits at Thomas Jefferson University Hospital and the Asian Pacific Liver Center. Data for the baseline survey were collected from August 2022 to July 2023. The Thomas Jefferson University Institutional Review Board approved this study. Informed consent was obtained from all individual participants included in the study.

Baseline Survey. After providing written consent, 365 CHB respondents completed the baseline activities, including hair sampling. Given the choice of language for the materials, 82% (n = 299) selected Korean, and 18% (n = 66) chose English. Most respondents (83%) opted for a self-administered modality (63% using an iPad on site, 26% using a remote link, and 11% completing a paper copy). Due to reading difficulties or discomfort with using an iPad, 17% of participants requested that the questionnaire be administered by an interviewer. The baseline survey took approximately 40 minutes to complete, and participants received a $50 gift card for their involvement.

As a part of a retrospective and prospective longitudinal study, the reported data included an analysis of the baseline assessments. The baseline survey encompassed sociodemographic data, social determinants of health, and quality of life measured by SF-12.

### Measures

Outcome. The SF-12 v2 consists of 12 items for assessing health-related quality of life (HRQoL) based on eight domains: physical functioning, role-physical, bodily pain, general health, vitality, social functioning, role-emotional, and mental health. All 12 items were used to calculate physical and mental component summary scores (PCS-12 and MCS-12) by applying a scoring algorithm empirically derived from the data of a US general population survey [36]. The PCS-12 and MCS-12 scores were generated and normalized based on a mean of 50 and a standard deviation of 10, with higher scores representing better health [34, 36].

The SF-12 summary descriptive statistics are presented in Table 1. Four of the items were recoded so that higher scores correspond to better health. Responses were clustered at the upper end of the measurement scale, as expected in a general population. Despite this, the full range of possible responses was used satisfactorily, supporting the overall sensitivity of the measurement model. The reliability and validity of the Korean version of the SF-12 were assessed [37]. In this study, the psychometric properties of the SF-12 were evaluated by exploratory factor analysis of principal component analysis (PCA) with varimax rotation. The two-factor solution had an explained variance of 61.7% including the PCS-122 (n = 6, alpha = .86) and MCS-12 (n = 6, alpha = .86).

Social Determinants of Health (SDOH) consists of five domains (see Table 1): First, education access and quality were measured by education (0 = < college graduate; 1 = more than college graduate) and survey language (0 = Korean; 1 = English). Second, economic stability was measured by employment (0 = no; 1 = yes), income (1=<$10K to 7=>$100K), and financial stability. Financial stability was measured by asking how your finances usually work out by the end of the month (1 = not enough to make ends meet; 2 = enough to make ends meet; 3 = some money left over). Third, the social and community context was measured by marital status (0 = not married; 1 = married) and social support. Social support was measured with an 8-item modified version of the Medical Outcomes Study Social Support (mMOS-SS) scale, measuring five levels of availability of key types of social support, from instrumental to emotional [(a) help if confined to bed, (b) take to the doctors, (c) prepare meals, (d) help with daily chores, (e) have a good time with, (f) advice on dealing with an issue, (g) understanding your problems, (h) providing love/feeling wanted]. Possible summed scores ranged from 0 to 32.

Fourth, the neighborhood and built environment were measured by perceived neighborhood social cohesion and neighborhood physical disorder. Neighborhood social cohesion. We used a previously validated scale, neighborhood social cohesion and physical disorder in the Project on Human Development in Chicago Neighborhoods Study [38], which has been used in multiple other studies [39, 40]. To assess neighborhood social cohesion, we first asked: “How often are these things a problem or are found in your neighborhood?” We then assess the following 4 aspects of neighborhood social cohesion: 1) People around here are willing to help their neighbors; 2) People in this neighborhood generally get along with each other; 3) People in this neighborhood can be trusted; and 4) People in this neighborhood share the same values. Response options included (1 = Strongly disagree to 5 = Strongly agree). A total neighborhood social cohesion score was calculated by summing these 4 items (Cronbach’s α = .85), ranging from 4 to 20 with a mean of 10.63 (SD = 3.16). To assess neighborhood physical disorder, 5 aspects of the neighborhood were assessed, including a lot of trash and litter on the street, a lot of noise, violence, attractiveness, and safe walking in the neighborhood. Response options included (1 = Strongly disagree to 5 = Strongly agree). A total neighborhood physical disorder score was calculated by summing these 5 items (Cronbach’s α = .81), ranging from 6 to 25 with a mean of 19.50 (SD = 4.67). Finally, healthcare access and quality were measured by having a PCP (0 = no; 1 = yes) and having health insurance (0 = no; 1 = yes).

*Covariates* were age and gender (0 = male; 1 = female).

#### Statistical Analysis.

First, descriptive statistics were used to consider sociodemographic characteristics, including frequency, mean, standard deviation, and range. Second, bivariate analysis using correlation analysis among all the variables was conducted to check the assumptions of multicollinearity. Then, linear regression models were performed to estimate associations between each domain of SODH and health outcomes with PCS12 and MCS12. Missing data was minimal (ranged from 0–7 out of 365). Statistical analyses were performed using STATA 17 (StataCorp, Texas, USA).

## RESULTS

Table 2 describes the characteristics of the cohort. The average age of 365 participants was 60.1 years (range 19–84, SD 10.7). The cohort was 44% female. Approximately 56% had completed college, and a third reported poor or no English proficiency. A majority (81%) were married or living with a partner, and about two-thirds were employed. The vast majority had some form of health insurance (93%) and a primary care provider (94%). The average time since HBV diagnosis was 26.9 years (range 5–52, SD 10.3), and 58% had a family history of HBV infection.

Table 3 presents zero-order correlations between variables. PCS-12 was correlated with four domains of SDOH, except for health care access, while MCS-12 was correlated with 4 domains of SDOH, except for education access and quality. Age was highly correlated with survey language (r = −0.48, P < 0.01): older individuals were less likely to use an English survey. All three of economic stability (e.g., employment, income, and financial stability) were highly correlated (r = .42, p < 0.01 for employment and income; r = 0.43, P < 0.01 for income and financial stability). Perceived neighborhood social cohesion and physical disorder were highly correlated (r = 0.45, P < 0.01). Income was also highly correlated with physical disorder (r = 0.47, p < 0.01). Thus, income and perceived physical disorder were excluded from the model to avoid multicollinearity.

Table 4 shows the results of multiple linear regression analyses of PCS-12 and MCS-12. Employment and financial instability were associated with quality of life: The Employed had higher scores of quality of life (β = 2.28, p = .009 for MCS-12; β = 1.76, p = .02 for PCS-12). Those with financial stability reported higher scores of quality of life: Compared to those with not enough, those with leftover (β = 2.38, p < .05) had higher MCS12. Those with enough (β = 4.32, p < .001) and leftover (β = 3.93, p < .001) had higher scores of PCS12 than those with not enough. Social support was positively associated with mental health (β = 1.68, p < .001). Perceived neighborhood social cohesion was also positively associated with mental health (β = 0.24, p < .05). In addition, females had lower levels of PCS-12 than males (β=−2.57, p < .001). Interestingly, age was negatively associated with PCS-12 and positively with MCS-12: the older reported the lower physical health (β=−.13, p = .002), while the older reported better mental health (β = .17, p < .001).

## DISCUSSIONS

This study examined how the five domains of SDOH related to HRQoL among Korean Americans living with CHB. Using the SF-12, we identified several key predictors of physical and mental health in this understudied population.

The SF-12 is widely used in both the general population and people with chronic conditions. The reliability and validity of the SF-12 in the Korean population have previously been reported [37]. However, no reports were found with summary statistics (e.g., mean, SD) on the Korean American CHB population. Notably, this cohort of CHB patients reported higher scores of PCS-12 and MCS-12 than stroke patients in Korea, a comparably vulnerable population [41]. This finding may suggest that despite living with a chronic viral infection, our patient cohort of Korean Americans in hepatology care may experience relatively preserved functional and mental health compared to other chronically ill populations.

Consistent with the previous studies [42, 43], economic stability (e.g., employment and financial stability) was positively associated with HRQoL. Financial security likely reduces stress, improves adherence to long-term antiviral therapy, and enhances access to care. Given the high lifetime treatment costs and prolonged disease duration in this cohort (mean 27 years), economic stability appears to play a central role in mitigating both physical and psychological disease burden.

Within social and community context, one of the SDOH domains, social support was associated with better mental health, which aligns with findings across other chronic disease populations [14–16]. In addition, marital status predicted better physical health, suggesting that interpersonal support may promote healthy behaviors and treatment adherence. However, marital status alone does not fully explain these differences; relationship quality, as well as race, ethnicity, and cultural factors, may influence this association [44]. Prior studies have shown that the protective effects of marriage vary by race, with stronger associations observed in Black adults than White adults [45]. Our findings suggest that, in the Asian American CHB population, marriage similarly contributes positively to physical health, which possibly reflects the cultural emphasis on family cohesion and caregiving within this community. Further investigation is needed to explore how relationship quality and cultural expectations moderate these effects.

The domain of neighborhood and built environment (e.g., perceived neighborhood social cohesion) was positively associated with mental health but showed a small, non-significant association with physical health. This complements prior work indicating that neighborhood cohesion can have dual effects: it may enhance psychosocial well-being through social support while simultaneously fostering contexts for behaviors that offset physical health benefits, such as reduced physical activity or unhealthy lifestyle habits. Such opposing mechanisms may cancel out observable effects on physical health [46].

Age- and gender-related differences in HRQoL in our study mirrored patterns previously reported in chronic disease populations. Older participants reported better mental health, consistent with prior studies on improved psychosocial well-being with aging [47]. This may reflect age-related increases in emotional regulation, mental resilience, and coping skills, which can reduce stress and enhance overall mental health. In contrast, our finding on physical health declined with age, which is likely due to increased comorbidities, higher prevalence of chronic conditions, and age-related functional decline [48]. Furthermore, female participants reported poorer physical health than males, possibly due to the combined effects of biological differences in pain perception, disproportionate caregiving roles, and gender-related disparities in healthcare access and utilization [49].

One of the strengths of this study is that it focused on Korean American patients with CHB who were receiving care from Korean American hepatologists, examining patient outcomes within a culturally and linguistically concordant care setting. Previous studies about HBV among Korean Americans have been limited to Korean Americans in the community or those receiving care in the general healthcare system [50, 51]. Another strength of the study is that our measure of health outcomes (PCS-12, MCS-12) was the gold-standard SF-12 scale—a widely used, valid, and reliable self-reported measure of quality of life in diverse populations [34]. Notably, this is the first study to use SF-12 among Korean Americans, whereas previous studies have used a single question of self-reported health, which, although widely used, may not be the most accurate measure. Moreover, simultaneous examination of all five SDOH domains outlined by one of the national priorities Healthy People 2030 initiative is unique. This study addresses calls from both Healthy People 2030 and the 2022 AASLD HBV-HDV Treatment Endpoints Conference, emphasizing the importance of assessing daily functioning and mental wellness alongside virologic outcomes [52].

Our study has several limitations. First, recruiting participants from only two clinical sites in Philadelphia and Los Angeles limits the generalizability of the results to all Korean American patients with CHB in the US. Second, reliance on self-reported data (e.g., English proficiency, SF-12) may introduce potential recall bias and inaccuracies.

Despite these limitations, this study has several important implications. Targeted interventions, including financial assistance programs, health insurance navigation support, may alleviate the financial stress associated with long-term antiviral treatment and thus improve treatment adherence. Community-focused programs, such as culturally tailored peer support groups, can enhance social connectedness, mitigate stigma, and promote healthy behaviors, especially in immigrant communities. Integrating SDOH screening into routine hepatology practice may also help to identify patients at risk of poor quality of life outcomes and enable timely referral to supportive resources.

Future research should employ longitudinal designs to examine how changes in social determinants influence HRQoL over time, investigate the interplay between biological indicators (eg. fibrosis scores, viral load, and laboratory markers) and patient-reported outcomes, and extend investigations to other Asian American subgroups. Furthermore, interventional studies are needed to evaluate the effectiveness of culturally tailored, community-focused programs in improving the overall quality of life among individuals living with CHB.

## Figures and Tables

**Table 1:** Five domains of SDOH

Domain	
Education Access and Quality	Education
	Survey language
Economic Stability	Employment
	Income
	Financial stability
Social and Community Context	Marital status
	Social support (n = 8, alpha = .96)
Neighborhood and Built Environment	Perceived neighborhood social cohesion (n = 4, alpha = .85)
	Neighborhood physical disorder (n = 5, alpha = .81)
Health Care Access and quality	Having health insurance
	Having PCP

**Table 2:** Characteristics of Korean American CHB patients (n = 365), 2021 to 2022

Variables%)	*N (%)*
Sociodemographic	
Age (mean ± SD, range)	60.09 ± 10.74 (19–84)
Gender	203 (55.6%)
Men	162 (44.4%)
Women	
Education	23 (6.4%)
<high school	136 (37.6%)
high school graduate+	133 (36.7%)
College graduate	70 (19.3%)
Graduate/professional school	
Spoken English proficiency.	96 (26.5%)
Fluent/well	146 (40.2%)
So so	121 (33.3%)
Poor/not at all	
Marital Status	295 (80.8%)
Married	70 (19.2%)
Not married (divorce/widow/separate/single)	
Employment (= yes)	227 (62.4%)
Having health insurance (= yes)	339 (92.9%)
Having PCP (= yes)	342 (94.2%)
Time since HBV diagnosis (year), (mean ± SD, range)	26.97 ± 10.33 (5–52)
Family history of HBV infection (= yes)	213 (58.4%)
Social support (mean ± SD, range)	29.50 ± 8.60 (8–40)

**Table 3:** Bivariate Analysis

		1	2	3	4	5	6	7	8	9	10	11	12	13	14
	**1.Age**	-													
	2.Gender	.09+	-												
I	3.Survey Language	− .48**	− .09+	-											
	4.Education	− .16**	− .12*	.27**	-										
II	5.Employment	− .40**	− .28**	.19**	.13*	-									
	6.Income	− .34**	− .14**	.30**	.29**	.42**	-								
	7.Financial stability	− .15**	− .08	.12*	.08	.26**	.43**	-							
III	8.Marital status	− .03	− .13*	− .04	.08	.19**	.33**	.13*	-						
	9.Social support	− .14**	− .07	.14*	.06	.10*	.22**	.17**	.29**	-					
IV	10.Social Cohesion	− .16**	− .14**	.24**	.08	.10+	.27**	.14**	.08	.18**	-				
	11.Neighborhood physical disorder	− .19**	− .17**	.32**	.25*	.18**	.47**	.22**	.16**	.25**	.45**	-			
V	12.Having health insurance	− .12*	− .08	.05	− .08	.01	− .02	.02	− .02	− .01	− .01	− .05	-		
	13.Having PCP	.19**	.03	− .22**	− .01	− .07	.02	− .01	.09+	− .02	− .07	− .06	− .11*	-	
	14.PCS-12	− .32**	− .28**	.23**	.19**	.33**	.29**	.23**	.22**	.17**	.17**	.23**	.05	− .06	-
	15.MCS-12	.15**	− .11*	− .10+	.01	.11*	.05	.21**	.11*	.23**	.12*	.10+	.02	.09+	.21**

Note.

+ p < .10;

* p < .05;

** p < .01. Gender (1 = male, 2 = female), survey language (1 = Korean, 2 = English), Education (1 = high school+, 2 = College+), employment (0 = no, 1 = yes), financial instability (1 = leftover, 2 = enough, 3 = not enough) Having health insurance (0 = no, 1 = yes), Having PCP (0 = no, 1 = yes)

**Table 4:** Multivariate Linear Regression Analysis of Quality of Life (n = 363)

		PCS12	MCS12
control	Age	− .13**	.18**
	Gender (1 = M; 2 = F)	−2.52**	− .68
I	Survey Language (1 = Korean; 2 = English)	.75	−1.50
	Education (1 = HS+, 2 = college+)	1.32	.28
II	Employment (0 = no; 1 = yes)	1.77*	2.28**
	Financial stability (0 = not enough)	1.84+	4.32**
	-enough	2.38*	3.93**
	-leftover		
III	Marital status (0 = not married; 1 = married)	2.62**	.03
	Social support	.03	.168**
IV	Perceived Neighborhood Social Cohesion	.11	.24*
V	Having health insurance (0 = no; 1 = yes)	.31	2.32+
	Having PCP (0 = no; 1 = yes)	.49	−2.07
R^2^		.248	.189

Note.

+ p < .10;

* p < .05;

** p < .01

## Data Availability

To protect participant privacy and confidentiality, the current study dataset is not publicly available. De-identified data may be made available from the corresponding author upon reasonable request.
